# Characterization and Modeling of Intermittent Locomotor Dynamics in Clock Gene-Deficient Mice

**DOI:** 10.1371/journal.pone.0058884

**Published:** 2013-03-13

**Authors:** Toru Nakamura, Toru Takumi, Atsuko Takano, Fumiyuki Hatanaka, Yoshiharu Yamamoto

**Affiliations:** 1 Graduate School of Education, University of Tokyo, Tokyo, Japan; 2 Graduate School of Biomedical Science, Hiroshima University, Hiroshima, Japan; 3 Research Center for Child Mental Development, Osaka University, Osaka, Japan; Kent State University, United States of America

## Abstract

The scale-invariant and intermittent dynamics of animal behavior are attracting scientific interest. Recent findings concerning the statistical laws of behavioral organization shared between healthy humans and wild-type mice (WT) and their alterations in human depression patients and circadian clock gene (*Period 2*; *Per2*) mutant mice indicate that clock genes play functional roles in intermittent, ultradian locomotor dynamics. They also claim the clinical and biological importance of the laws as objective biobehavioral measures or endophenotypes for psychiatric disorders. In this study, to elucidate the roles of breakdown of the broader circadian regulatory circuit in intermittent behavioral dynamics, we studied the statistical properties and rhythmicity of locomotor activity in *Per2* mutants and mice deficient in other clock genes (*Bmal1*, *Clock*). We performed wavelet analysis to examine circadian and ultradian rhythms and estimated the cumulative distributions of resting period durations during which locomotor activity levels are continuously lower than a predefined threshold value. The wavelet analysis revealed significant amplification of ultradian rhythms in the BMAL1-deficient mice, and instability in the *Per2* mutants. The resting period distributions followed a power-law form in all mice. While the distributions for the BMAL1-deficient and *Clock* mutant mice were almost identical to those for the WT mice, with no significant differences in their parameter (power-law scaling exponent), only the *Per2* mutant mice showed consistently and significantly lower values of the scaling exponent, indicating the increased intermittency in ultradian locomotor dynamics. Furthermore, based on a stochastic priority queuing model, we explained the power-law nature of resting period distributions, as well as its alterations shared with human depressive patients and *Per2* mutant mice. Our findings lead to the development of a novel mathematical model for abnormal behaviors in psychiatric disorders.

## Introduction

Mental or cognitive brain functions and their alterations in psychiatric diseases are difficult to approach through biological techniques because of lack of appropriate assay systems with objective measures. Locomotor activity can be measured objectively in virtually any animal, and its alteration in humans is one of the cardinal signs of psychiatric disorders [1]. Beyond traditional measures such as mean activity levels, the focus is therefore on the dynamical properties of locomotor activity, aiming for more objective and quantitative measures of the type of activity disturbances observed in human neurobehavioral disorders.

Recently, the scale-invariant and intermittent properties of animal behavioral dynamics, including their mathematical modeling, have attracted broad scientific interest [2], [3], [4], [5], [6], [7], [8], [9]. For example, the statistical laws of behavioral organization, specifically those describing the statistical properties of durations of resting and active periods derived from locomotor dynamics in daily life, have been shown to be identical between healthy humans and wild-type (WT) mice [10]. The durations of active periods during which physical activity levels (wrist acceleration counts for humans and integrated force platform data for mice) are continuously above a predefined threshold, when rescaled with respect to individual means, follow an identical stretched exponential cumulative distribution. On the other hand, the durations of resting periods below the threshold obey a power-law cumulative distribution with identical parameter values for both these mammalian species. Furthermore, the alteration in such a shared statistical law of resting period distributions (a significant decrease in power-law scaling exponents) among humans with major depressive disorders, mice with a functional deficiency in a circadian clock gene (*Period 2*; *Per2*) [10], [11], and patients with schizophrenia [12] has been confirmed. This indicates the increased intermittency, characterized by reduced activity associated with occasional bursts, in ultradian or within-day locomotor dynamics in the *Per2* mutant mice and human psychiatric patients.

These findings indicate that the common or *universal* laws of behavioral organization have values that are interpretable both clinically and biologically as objective biobehavioral measures or endophenotypes for psychiatric disorders, and the study of behavioral organization could provide further insight into the pathophysiology of psychiatric disorders and contribute to the development of their animal models. In addition, the alteration in the statistical laws in the *Per2* mutant mice might suggest that circadian clock genes have some functional role in organizing intermittent locomotor dynamics.

In the present study, we questioned whether such characteristic locomotor dynamics and an interesting biobehavioral endophenotype characterizing human depression are associated with breakdown of the broader circadian regulatory circuits. We thus studied the statistical properties of locomotor activity, along with the circadian and ultradian rhythmicity, of the *Per2* mutant mice and mice deficient in other core clock genes (*Bmal1*, *Clock*). Furthermore, we mathematically explain a possible mechanism for power-law nature of resting period distributions, as well as its alterations shared with human depressive patients and *Per2* mutant mice, using a stochastic priority queuing model.

## Results

### Alterations in Circadian and Ultradian Rhythms

**Figure 1:**
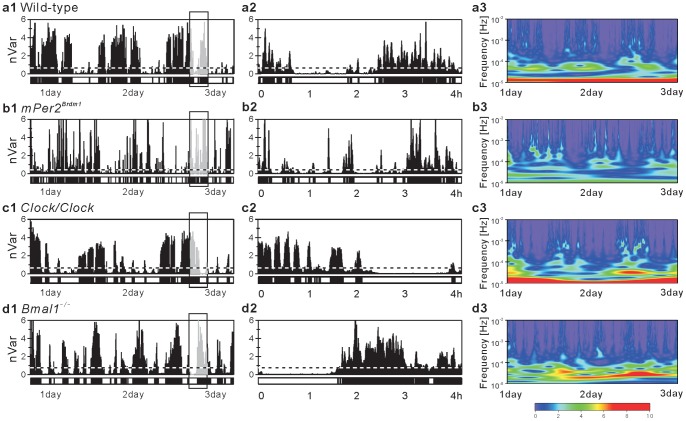
Fluctuation of locomotor activity. Illustrative examples of locomotor activity data (local variance with window size 60 s) of a (**a**) WT, (**b**) *mPer2^Brdm1^*, (**c**) *Clock/Clock*, and (**d**) *Bmal1^−^* ^/−^ mouse over 3 consecutive days (left panels). The middle panels are magnifications of the left panels with 4-h periods during subjective daytime. The overall average of non-zero activity levels is used as a threshold (horizontal dotted line), and the period during which the levels are continuously below or above the threshold is coded as a resting (open bar in bottom panels) or active (closed bar) period, respectively. Because body weight varies across individuals, activity levels in the vertical axis (nVar) are normalized by the mean variance of each record. The right panels are the moduli of CWT of locomotor activity shown in the left panels. Values of moduli are color coded according to their magnitude (blue indicates a low and red a large value), and the ordinates are represented on a logarithmic scale.

**Figure 2:**
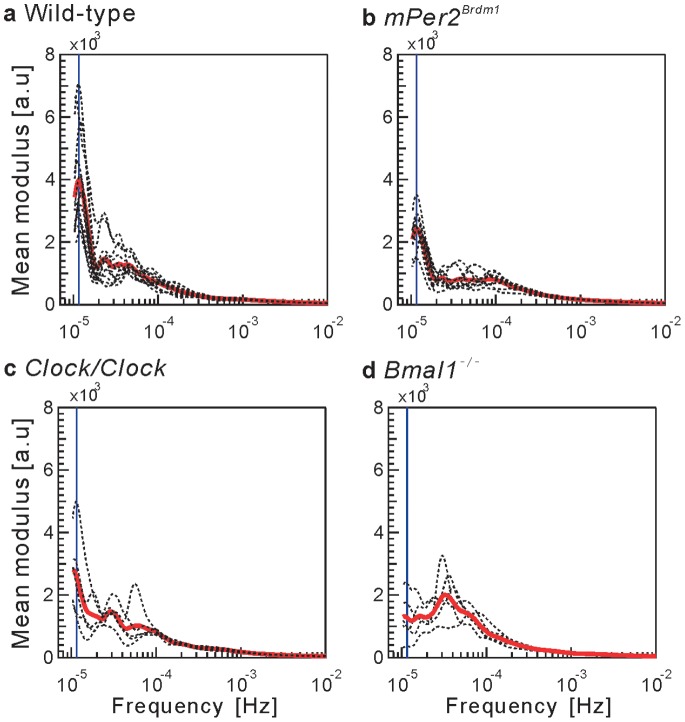
Spectrum density of locomotor activity. Group-averaged spectrum estimated from the CWT modulus for (**a**) WT, (**b**) *mPer2^Brdm1^*, (**c**) *Clock/Clock*, and (**d**) *Bmal1^−^* ^/−^ mice on a semi-logarithmic scale (thick solid red curves). The spectrum of each mouse (broken curve) is superimposed. The vertical blue line corresponds to 1.2×10^−5^ Hz (approximately 24 h).

Figure 1 shows the representative patterns of locomotor activity in our mice. Mutation or knockout of a circadian clock gene is well known to alter the circadian rhythm of locomotor activity. Consistent with previous reports [13], [14], [15], we confirmed the disrupted patterns of the circadian rhythm in our mice. Mutation in the *Per2* gene, providing a negative feedback loop in the circadian regulatory circuit [15], shortens the circadian cycle and then gradually leads to arrhythmic locomotor patterns such as those seen in Fig. 1(b1). These alterations can be confirmed by the spectrum of locomotor activity in the *Per2* mutant mice, such as the shifted circadian peak toward higher frequency ranges (23.1±0.2 h) and the significant decrease in the magnitude of the circadian component compared with the WT mice [Fig. 2(b) and Table 1]. Mutation in another circadian clock gene, *Clock* [14], lengthens that cycle [Fig. 1(c1)], with a significant shift of circadian peaks toward significantly longer frequency ranges (27.7±1.2 h) [Fig. 2(c) and Table 1]. Deficiency in *Bmal1*, which is critically important for the generation of the circadian oscillation [13], completely disrupts the circadian rhythm and immediately leads to arrhythmic behavior [Fig. 1(d1)]. This property is reflected in the extinction of the circadian peak in the spectrum [Fig. 2(d)].

**Table 1:** Comparison of circadian and ultradian rhythms.

	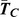 [hrs]	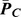	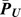	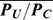
Wild-type	24.0±0.3	2401.4±261.1	1005.5±57.0	0.46±0.04
*mPer2^Brdm1^*	23.1±0.2	**1550.8±116.7** *	795.2±42.7	0.53±0.05
*Clock/Clock*	**27.7±1.2** *	1724.2±418.3	1015.3±131.8	0.72±0.18
*Bmal1* ^−/−^	–	**1254.2±240.0** *	**1340.1±106.9** *	**1.19±0.17** *

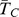
: circadian rhythm, 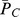: area of circadian component (a.u: arbitrary unit), 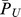: area of ultradian component (a.u), 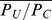: ratio of ultradian to circadian component. Values are shown as mean ± SEM.

* indicates the significant difference from WT mice (Dunnett’s multiple comparison, *p*<0.05). Note that *Bmal1* ^−/−^ mice were excluded from the group comparison of 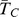 due to the extinction of the circadian rhythm (refer to Results).

The significant peaks of ultradian rhythms were distributed in the range 1.88×10^−5^–4.53×10^−5^ Hz (approximately 6.1–14.8 h) in the WT mice, while the number of peaks and their dominant frequency were not consistent across individuals [Fig. 2(a)]. In contrast, the *Bmal1* ^−/−^ mice showed significant amplification of the spectrum at approximately 3.24×10^−5^ Hz (approximately 8.6 h) [Fig. 2(d)], and the contribution of this rhythmicity in fluctuation of locomotor activity was the most dominant (Table 1). The *Per2* mutant mice were characterized by both decreasing tendency and flatter shape of the group-averaged spectrum of the ultradian component [1.99×10^−5^–9.84×10^−5^ Hz (approximately 2.8–9.8 h)], suggestive of instability and/or disruption of the ultradian rhythm [Fig. 2(b) and Table 1].

### Alterations in Statistical Laws of Behavioral Organization

**Figure 3:**
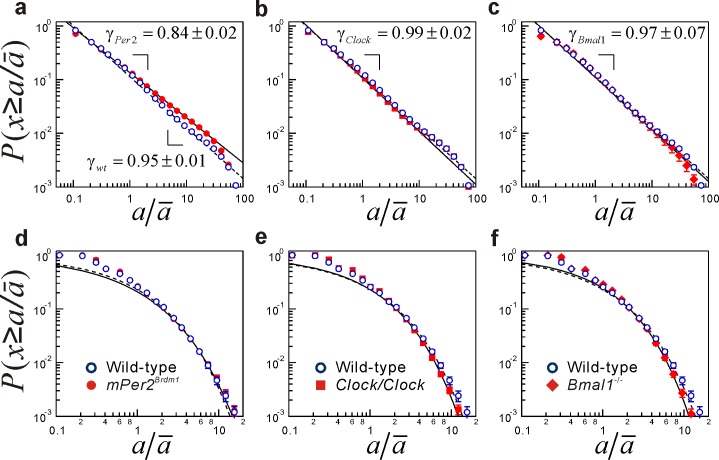
Cumulative distributions of resting and active period durations in clock gene-deficient mice. (**a**)–(**c**) Comparison of group-averaged cumulative distributions 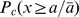 of rescaled resting period durations 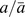 between the WT mice and (**a**) *mPer2^Brdm1^*, (**b**) *Clock/Clock*, and (**c**) *Bmal1^−^* ^/−^ mice, where a non-zero mean is used as the threshold value. Error bars indicate standard error of the mean. Straight lines are eye guides with the group mean parameter values (WT, 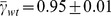; *mPer2^Brdm1^*, 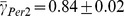; *Clock/Clock*: 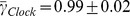; *Bmal1* ^−/−^, 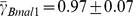). (**d**)–(**f**) Group-averaged cumulative distributions of active period durations. The broken (WT mice) and solid (deficient mice) curves are stretched exponential functions with the group mean parameter values shown in Table 2. Note that the resting distributions are shifted along the vertical axis to take the same value at 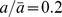, and the distributions are plotted with wider bins (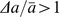) for the purpose of illustration. The results for the WT and *mPer2^Brdm1^* mice are reproduced from our previous study [10].

Figure 3 shows the comparisons of rescaled cumulative distributions of the durations of resting [Fig. 3(a)–(c)] and active periods [Fig. 3(d)–(f)] between the WT and clock gene-deficient mice, where a non-zero mean is used as a threshold value. In all mice, the resting period distributions had a power-law form for more than two decades (from 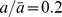
–20). On the other hand, the active period distributions were well approximated by a stretched exponential functional form over the range from 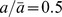
–10. Consistent with our previous work [10], the mean scaling exponent 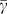 of resting period distributions in the *Per2* mutant mice was significantly smaller than that in the WT mice [reproduced in Fig. 3(a) and Table 1 for the comparison]. This significant decrease in 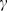 is confirmed by the fatter tail in a longer duration range than that for the WT mice, suggestive of a systematic increase in resting events with longer durations or increased intermittency of locomotor activity. This is also supported by the increasing tendency of mean resting durations 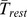 (Table 2). In contrast, the other clock gene-deficient mice (*Clock/Clock* and *Bmal1* ^−/−^) did not show any significant difference from the WT mice in the scaling exponents (Table 2), regardless of the distinct disruption of their circadian rhythms. These findings may indicate that the circadian rhythm is not the principal factor leading to the alteration in resting period distributions. The stretching exponent *β* of active period durations was not significantly different among groups.

**Table 2:** Comparisons of behavior organization parameters.

	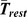 [sec]	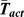 [msec]	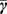	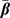
Wild-type	1.89±0.18	361.3±16.7	0.95±0.01	0.59±0.03
*mPer2^Brdm1^*	2.14±0.29	344.3±18.9	**0.84±0.02** *	0.55±0.03
*Clock/Clock*	1.50±0.08	317.4±8.2	0.99±0.02	0.62±0.03
*Bmal1* ^−/−^	1.23±0.18	325.1±33.0	0.97±0.07	0.65±0.03

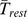
: mean duration of resting periods, 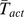: mean duration of active periods, 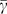: mean scaling exponent of rescaled cumulative distributions of resting periods, 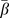: mean stretching exponent of rescaled cumulative distributions of active periods. The values, where non-zero mean is used as a threshold value, are shown as mean ± SEM.

* indicates the significant difference from WT mice (Tukey post-hoc test with Sidak adjustment for the multiple comparison, *p*<0.05). The results for WT and *Per2* mutatnt mice are reproduced from our recent study [10].

**Figure 4:**
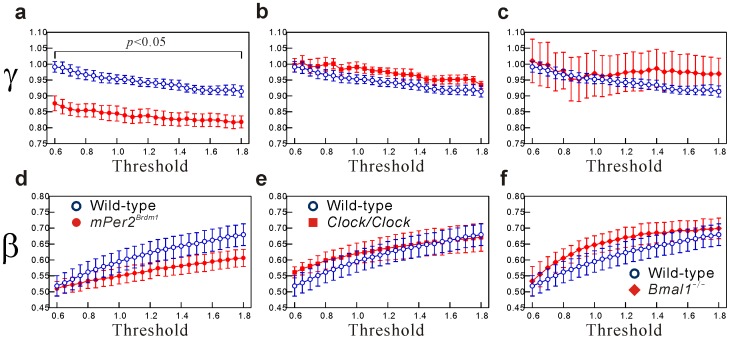
Dependency of distribution parameters on threshold values. The values of *γ* with different threshold values of 0.6, 0.7, …, 1.7, and 1.8 times the overall average for the non-zero mean for (**a**) *mPer2^Brdm1^*, (**b**) *Clock/Clock*, and (**c**) *Bmal1^−^* ^/−^ mice. (**d**)–(**f**) The same as (**a**)–(**c**) but for *β*. Bars indicate standard error of the mean.

To confirm the robustness of our findings, we examined the effects of threshold values on the distribution parameters. Specifically, for the locomotor activity, we used various threshold values ranging from 0.6 to 1.8 times the overall mean of non-zero activity levels and evaluated the distribution parameters. As shown in Fig. 4, the resultant estimate 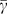 of resting period distributions in the *Per2* mutant mice was consistently and significantly smaller than that in the WT mice [Fig. 4(a)], while the others were not significant at all threshold values examined. These findings indicate the robustness and consistency of our results.

## Discussion

We investigated alterations in locomotor dynamics in clock gene-deficient mice and demonstrated the characteristic disruptions in both circadian and ultradian rhythms in their locomotor activity. However, apart from these dysfunctions in rhythmicity, locomotor activity in all the deficient mice followed the same statistical laws of behavioral organization. In addition, the alteration in the statistical laws of resting periods was specific to the *Per2* mutant mice, showing the increased intermittency in ultradian locomotor dynamics (i.e., decreased 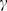), an endophenotype for immobility shared among some psychiatric disorders, such as depression [10], [11] and schizophrenia [12].

### Relationships between Circadian Clock Genes and Ultradian Rhythms

Previous studies on mice with deficiency in *Period 2* [15], *Clock* [14], and *Bmal1* [13] genes demonstrated the existence of ultradian rhythmicity with periods ranging from 4–12 h (*mPer2^Brdm^*, 4–8 h; *Clock/Clock*, 6–9 h; *Bmal1* ^−/−^, 5–12 h) under the constant dark condition. These authors speculated that the ultradian locomotor rhythms might not be driven by the circadian oscillatory system because animals with a lesion of the suprachiasmatic nucleus were reported to maintain ultradian rhythmicity [16], [17], [18], [19]. Our results are generally compatible with these findings in that, while spectral analysis revealed significant effects of clock gene mutations on the circadian component, the ultradian rhythms are relatively well maintained. However, our systematic and robust analysis with high-resolution data measured under the same conditions further revealed that only the *Per2* mutant mice tend to show instability in ultradian locomotor dynamics, manifested by the flat and diminished spectral power in the corresponding range [Fig. 2(b)]. In addition, these mutant mice showed alterations in the statistical laws of behavioral organization characterizing ultradian dynamics with more higher-frequency. The functional roles of the *Per2* gene, providing a negative feedback loop in the circadian regulatory circuit [15], in controlling ultradian rhythms need further study.

### An Aspect of Dysfunction in Neurotransmitter Systems

The reason why the increased intermittency in ultradian locomotor dynamics is found only in the *Per2* mutant mice is unknown, but neurological and behavioral similarities that have been found between human psychiatric patients and the *Per2* mutant mice would be helpful in understanding our findings. The prevalence of comorbidity of psychiatric disorders with alcohol and drug abuse is known to be high [20], suggesting the involvement of common neurotransmitter systems, such as the dopaminergic and glutamatergic systems, in both cases. In mice, functional roles of circadian clock genes in neurotransmitter systems have been reported recently [21], [22]. For example, mutation in the *Per2* gene alters the glutamatergic system and then leads to hyperglutamatergic states of the brain, resulting in increased preference for and voluntary consumption of alcohol [23], a common behavior in human depression patients [20]. This dysfunction in the glutamatergic system could be understood as an alteration in the reinforcement system (i.e., the reward system) driving enhanced motivation to consume alcohol.

Furthermore, it has been demonstrated that such excessive alcohol consumption is reduced by treatment with acamprosate, a glutamatergic *N*-methyl-*D*-aspartate (NMDA) antagonist commonly used in clinics to prevent relapse in alcoholics. Interestingly, another NMDA antagonist, ketamine, has recently received considerable attention as an agent producing a rapid antidepressant effect in depression patients [24], [25], supporting the idea that the glutamatergic system plays an important role in the pathophysiology and treatment of mood disorders. In addition, the *Per2* mutant mice exhibit drug abuse-related behavior, such as enhanced sensitization in response to repeated cocaine admission [26], probably related to dysfunction of the reward system. We speculate that these similarities, especially the alterations in the brain’s reward systems related to dysfunction of neurotransmitter systems, might partly explain why the *Per2* mutant mice exhibited an alteration in ultradian locomotor dynamics, as also observed in human depression.

### Modeling Perspective

One of the important features of our analysis of behavioral organization is that it is possible to provide a theoretical explanation for altered behavioral dynamics not only in human psychiatric disorders [11], [12] but also in animal models [10]. We suggest that such a universal mechanistic view would help elucidate the underlying causes of neurobehavioral disorders and develop a novel mathematical model for them. Thus, we further study the alteration in resting period distributions in the *Per2* mutant mice based on a mathematical model.

Most animal activities, including slight movements, are considered to be triggered by continuously presented internal or external demands and/or stimuli; on the basis of the biological importance of the stimuli, animals are required to decide either consciously or unconsciously which stimuli they should respond to. The question arises as to what decision-making strategy is optimal. One possible strategy–and the simplest–is highest priority selection or the deterministic choice of a demand with the highest biological priority. In this case, the animal would always decide to respond to the most important demand, e.g., appetite, and seldom choose others, e.g., emotion or exploration; note that the demand with highest priority can either be internal or external, depending on a given situation. An alternative strategy is that each demand is probabilistically chosen every time in proportion to its biological importance. In this case, a demand with a lower priority is selected more frequently, leading to a wider variety of behaviors. As shown below, we found that, while the latter strategy is compatible with the actual statistical properties of resting periods in WT, the *Per2* mutant mice show the departure from the proportional toward highest priority selection.

**Figure 5:**
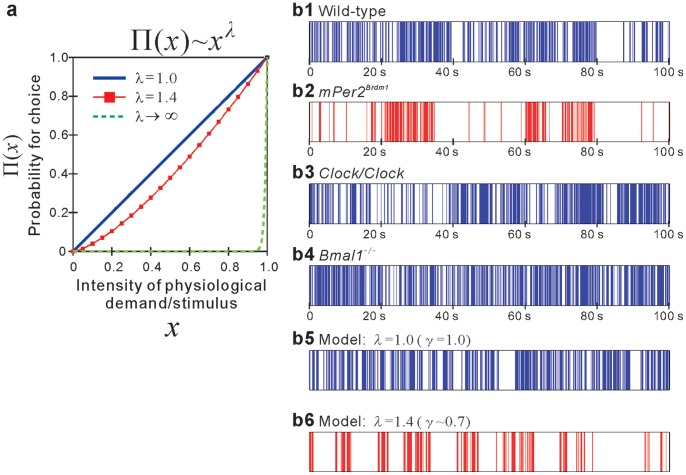
Sequence of resting period durations and the model. (**a**) Probability density function 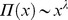 for choosing a demand/stimulus with priority *x*. The sequence of onset of activity bursts (resting durations) derived from locomotor activity of a (**b1**) WT (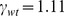), (**b2**) *mPer2^Brdm1^* (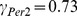), (**b3**) *Clock/Clock* (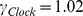), and (**b4**) *Bmal1^−^* ^/−^ mouse (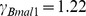) for 100 s. Note that these mouse data were calculated from locomotor activity shown in Fig. 1. (**b5**) The sequence of waiting times simulated from the priority stochastic queuing model with 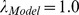 (i.e., 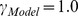) and (**b6**) 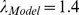 (i.e., 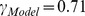). The simulated sequences of waiting time were generated on the base of the stochastic priority queuing model [2] with a priority list comprising *L* = 10 demands, where a priority parameter *x_i_* (*i = *1, …,*L*) chosen from a uniform distribution 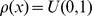 is assigned to each demand. At each time step, one demand is selected from the list (in the brain) according to 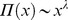 for execution (or act), and then removed from the list. At that moment, a new demand is added to the list with a priority randomly selected from 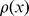. The probability that a demand with priority *x* is executed at time *t* is given by 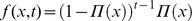, and the average waiting time of a demand with priority *x* is obtained by averaging over *t* weighted with 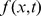, giving rise to 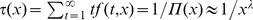. Analytically, with conservation law of probability (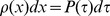), the waiting time distribution of the demands is given by 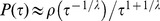. Note that each vertical line separates the successive waiting time of demands chosen according to their priority.

Mathematically, with the probability of response to a demand with priority *x* given by 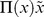 [Fig. 5(a)], the cumulative distribution of durations of resting periods is analytically derived to follow the power-law functional form with the exponent 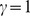 [2], which is commonly observed in locomotor activity in WT (as well as *Clock/Clock* and *Bmal1* ^−/−^) mice and healthy humans [10], [11]. Indeed, the model with this assumption nicely reproduces the sequence of onset of activity bursts [Fig. 5(b5)], which is similar to that of the WT mice [Fig. 5(b1)] and other clock gene-deficient mice (*Clock/Clock* and *Bmal1* ^−/−^) [Fig. 5(b3) and (b4)].

In contrast, the alteration in durations of resting periods in the *Per2* mutant mice is reproduced by assuming 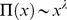 with 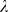 greater than unity (e.g., 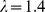). This assumption implies that a demand with higher priority is preferentially selected [Fig. 5(a)], giving rise to a fatter tail of resting period distributions (i.e., decreased 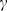) analytically [2] and experimentally in human depression [10], [11]. Indeed, the model with this condition generates a more intermittent sequence of onset of activity bursts [Fig. 5(b6)] observed in the *Per2* mutant mice [Fig. 5(b2)]. Such a strategic change in decision-making–preferential selectivity to demands with higher priority–may be related to reinforcement of rewarding neural networks induced by dysfunction of the dopamine and/or glutamatergic systems, as discussed in the previous section.

It is of note that the model with deterministic choice has already been rigorously studied by Barabási et al. [2], [27], showing that the stochastic priority queuing model successfully accounted for the heavy-tailed distributions of waiting time statistics observed in human social activities, such as e-mail communications, web browsing, and trade transactions, by assuming 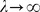 in 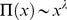. Different from human social activities, we suggest that the model with proportional choice is more appropriate to the characterization of animal behavioral organization.

### Conclusion

We suggest that the behavioral organization of locomotor dynamics, especially intermittent and complex dynamics, has interpretable biological value as objective biobehavioral measures or endophenotypes for psychiatric disorders, and its study could provide further insight into the pathophysiology of psychiatric disorders and contribute to the development of their animal and even mathematical models.

## Materials and Methods

### Assessment of Locomotor Activity in Mice

Locomotor activity data were acquired from mice with mutations and functional deficiency in three different types of circadian clock genes (*Bmal1* ^−/−^, *n = *5; *Clock/Clock*, *n = *5; and *mPer2^Brdm1^*, *n = *9) and WT mice (male C57BL6/J, *n = *12) over 3 consecutive days. *Bmal1* ^−/−^ mice [13] (originally generated by Dr. Chris Bradfield, University of Wisconsin Madison) were provided by Dr. Tohru Minamino (Chiba University Graduate School of Medicine). *Bmal1* ^−/−^ mice backcrossed with the C57BL/6J strain for 13 generations were intercrossed to generate WT and homozygous mutant mice. A breeding colony of homozygous *Clock* mutant mice [14] on a BALB/c background was developed using mice originally supplied by Dr. J. S. Takahashi (Northwestern University). Genotypes were determined for each individual by a PCR mutagenesis method before surgery. *mPer2^Brdm1^* (C57BL/6-Tyr^C−brd^) mice were provided by the Jackson Laboratory [15]. All mice were housed in cages in a 12 h light:12 h dark cycle, with access to food and water *ad libitum*. After more than 10 days of entrainment, the mice were placed in constant darkness and data were obtained for 3 consecutive days. The experimental procedures and housing conditions for animals were approved by the Animal Research Committee of Osaka Bioscience Institute and the Committee of Animal Experimentation, Hiroshima University.

The measuring and pre-processing procedures were essentially the same as those used in our previous work [10]. A piezoelectric sensor sheet placed under the cages was used to measure the daily activity of the mice. The sensor sheet produces a voltage signal that is proportional to the pressure generated by the mice’s activity. The signal was sampled at 100 Hz with 16-bit resolution after passing through a band-pass filter at 0.5–50 Hz. We divided the sensor data into intervals (windows) of equal length. In each window, we fitted a linear function that represented the trend in that window. Then, after subtracting the local trend in each window, the local variance (energy) of the detrended sensor data was calculated. The time series of the local variances obtained were regarded as the locomotor activity data of the mice (Fig. 1).

When evaluating the frequency characteristics of locomotor activity, we constructed time series with the window size of 60 s to reduce the computational cost and to focus on the ultradian locomotor dynamics on a time-scale of minutes to hours. In fact, we examined locomotor periodicity less than 60 s using a much smaller window size, but we did not find significant periodicity within this time-scale. In contrast, for the statistical laws of behavioral organization governing the locomotor dynamics on a much faster time-scale (within a few minutes) [10], we used the locomotor activity data with a window size of 0.1 s. However, the statistical laws of behavioral organization were previously shown to be robust against the choice of the window size from 0.01 s to 10 s [10].

### Evaluation of Circadian and Ultradian Rhythms

We used spectral analysis to study the effects of mutation in circadian clock genes on circadian and ultradian rhythms in locomotor activity. Because of the intermittent and non-stationary nature of locomotor activity in clock gene-deficient mice, we adopted spectral analysis on the basis of the continuous wavelet transform (CWT) defined below [28], [29]. CWT of a signal 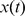 is defined as the convolution of the signal with a scaled and translocated basic function 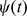:
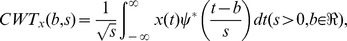
where * indicates the complex conjugate. CWT is a multiresolution approach that can decompose a signal into the time–frequency domain. The translocation parameter *b* allows us to focus on a particular part of the signal in time for analysis. The choice of the scaling parameter *s* changes the frequency content of the basic function; with small values of *s*, the basic function distributes in a narrower space in the frequency domain and thus corresponds to a higher frequency. With large values of *s*, it corresponds to a lower frequency.

The Morlet wavelet was chosen as a basic function for CWT. It has the following form:
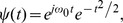
where 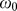 is a non-dimensional frequency. Here we used 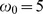 for the analysis. Because this wavelet has a fast-decaying oscillating waveform in the time domain and is compact in the frequency domain, it is efficient in extracting a periodic pattern from a signal. The relation between scale *s* and frequency 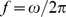 (Hz) is given by [29]



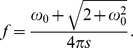



In this study, we calculated the wavelet transform from 10^−2^ to 10^−5^ Hz (approximately 100 s–27 h).

To obtain the spectrum of locomotor activity, we calculated the modulus of the wavelet coefficient defined by
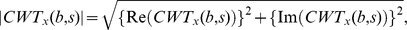
where 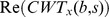 and 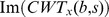 are the real and imaginary parts of the wavelet transform, respectively. The normalized spectrum was estimated by integrating the moduli over the entire time at each scale (frequency) and then dividing it by the total area, ranging from 10^−5^ to 10^−2^ Hz so that the corresponding area became unity.

From the estimated spectrum, we identified the period of the circadian rhythm as the location of the most dominant peak closest to 24 h. In addition, we evaluated the area (contribution) of the circadian component 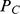 [1.0×10^−5^–2.0×10^−5^ Hz (approximately 14.1–26.8 h)] and ultradian component 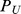 [2.0×10^−5^–1.1×10^−4^ Hz (approximately 2.6–14.1 h)] by integrating it. We also evaluated the ratio 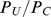.

### Cumulative Distributions of Resting and Active Periods

We estimated the cumulative probability distribution 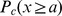 of durations *a* of resting periods during which locomotor activity levels are continuously lower than a certain predefined threshold value. We also estimated 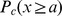 of durations *a* of active periods during which the activity levels are continuously above the threshold [10], [11], [12]. The cumulative probability distributions were obtained by numerically integrating the probability density function 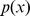 estimated from the whole recording period with a bin width of 0.1 s as follows: 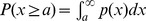. Rescaled cumulative distributions 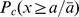 were also estimated from the rescaled probability density function, where both durations of resting and active periods were respectively rescaled by individual means 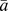.

Following our previous work [10], [11], [12], we fitted a power-law form 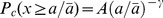 to the rescaled cumulative distributions of resting periods and a stretched exponential functional form 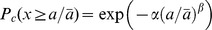 to those of active periods on the basis of a local minimum optimization of the chi-square statistic 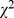 using the orthogonal distance regression and Levenberg–Marquardt minimization algorithm. We set the fitting range as 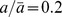
–20 and 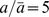
–10 for the cumulative distributions of durations of resting and active periods, respectively.

By definition, the choice of threshold values affects the durations of resting and active periods (the higher the threshold, the longer the mean resting period 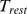 and the shorter the active period 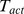). Therefore, we used different threshold values for the locomotor activity data and then examined the effect of the threshold values on the distribution parameters.

### Statistics

One-way ANOVA followed by Dunnett’s multiple comparison as a post hoc test was used to compare the mean values of the circadian and ultradian parameters. To analyze behavioral organization parameters, repeated measures one-way ANOVA with Tukey post hoc test was performed. The significance level was corrected using Sidak adjustment for multiple comparisons. 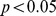 was considered significant.
